# An Evaluation of the Radioactive Content of Ashes Obtained from the Use of Fuels from Recycled Materials by Co-Processing in the Cement Industry

**DOI:** 10.3390/ma17102287

**Published:** 2024-05-12

**Authors:** José Antonio Suarez-Navarro, Miguel Ángel Sanjuán, Pedro Mora, María del Mar Alonso

**Affiliations:** 1 Department for Environment, Environmental Radioactivity and Radiological Surveillance Unit, CIEMAT Research Center of Energy, Environmental and Technology, Av. Complutense, 22, 28040 Madrid, Spain; ja.suarez@ciemat.es; 2 Department of Building Materials, Civil Engineering School, Technical University of Madrid, Profesor Aranguren, s/n, Ciudad Universitaria, 28040 Madrid, Spain; 3 Department of Geological and Mines Engineering, Mine and Energy Engineering School, Technical University of Madrid (UPM), C/Ríos Rosas, 21, 28003 Madrid, Spain; pmora@oficemen.com; 4 Eduardo Torroja Institute for Construction Sciences (IETcc-CSIC), 28033 Madrid, Spain; mmalonso@ietcc.csic.es

**Keywords:** recycled materials, co-processing, principal component analysis, cement, gamma spectrometry, XRF

## Abstract

The co-processing of different wastes as fuels in the manufacture of cement clinker not only meets the objectives of a circular economy but also contributes to the reduction in CO_2_ emissions in the manufacture of Portland cement. However, waste used as alternative fuels, such as sludge or organic-rich residues, may contain naturally occurring radionuclides that can be concentrated during the combustion process. In this study, the presence of natural radionuclides (radioactive series of uranium, thorium, and ^40^K) and anthropogenic radionuclides (^137^Cs) in these wastes has been investigated by gamma spectrometry. Possible relationships between the radioactive content and the obtained chemical composition, determined by X-ray fluorescence, have also been studied by applying a principal component analysis (PCA). The results showed that the wastes with the highest radioactive content were sewage sludge with activity concentrations of ^238^U and ^210^Pb of 321 ± 38 Bq kg^−1^ and 110 ± 14 Bq kg^−1^, respectively. A correlation between radioactive content and Fe_2_O_3_ concentration was also observed. The annual effective dose rates to workers for the ashes estimated from the ash content ranged from 0.0033 mSv to 0.092 mSv and therefore do not pose a risk to workers as they are lower than the 1 mSv per year limit for the general public (DIRECTIVE 2013/59/EURATOM).

## 1. Introduction

In the cement sector, as in other productive sectors, co-processing aims to promote sustainability by efficiently using waste materials, contributing to environmental protection, resource conservation, landfill reduction, and cost savings [1]. In fact, co-processing is determined when there is a dual material and energy benefit from waste in the same operation. Co-processing can be quantified by the recycling rate, defined as the proportion of waste materials recycled instead of being disposed of by usual routes such as landfilling or incineration [2]. The recycling rate plays an essential role in promoting sustainable waste management practices, reducing the environmental impact of waste, and moving towards the targets of the circular economy by encouraging the reuse and recycling of materials to minimize waste production.

In the manufacture of cement clinker, the percentage of recyclable materials from solid recovered fuels (SRFs) during co-processing in the cement industry can range from 76.8% to 100% [3]. This percentage represents the SRF ash that is considered recycled after incorporation into the clinker of Portland cements. SRF ashes containing non-combustible minerals, which are necessary for the manufacture of clinker, are integrated during the thermal recovery process. For this reason, SRF ash becomes a secondary raw material for the manufacture of cement clinker. The SRF is determined from the determination of the oxides in the SRF ash obtained by analytical techniques such as XRF or ICP-OES, which allows the recycling rate to be evaluated [4]. For this purpose, the R-index is used, which is given by the following expression [5]:

(1) $$R\text{-}index=\frac{AC}{100}\cdot \left(\omega_{1}+\omega_{2}+\cdots +\omega_{n}\right)$$

where *AC* is the ash content (wt%_DT_) and $\omega_{n}$ is the mass share of the oxides in the ash (wt%_DT_). Although there are different criteria to determine the *R-index*, the most accurate value is the one determined from nine oxides, Al_2_O_3_, CaO, Fe_2_O_3_, K_2_O, MgO, Na_2_O, SO_3_, SiO_2_, and TiO_2_, which will be indicated with the term R-index_9_[(%d)] [1].

The materials used in co-processing in the cement industry can be from a number of different origins [2]: urban (UW) and industrial waste (IW), meat and bone meal (MBM), sewage sludge (STPS), end-of-life vehicle waste (ELVM), wood (WD), etc. The few existing studies on the potential hazardousness of these materials are based on the determination of heavy metals. Xu et al. [3] highlighted the need for control of this waste in China due to the presence and release of heavy metals, such as Cr and Pb. From a radiological protection point of view, urban wastes (UWs) have been studied in different incineration plants [4], and it has been determined that, in general, they do not represent a risk for workers or the general population. The presence of natural radionuclides and the radioactive content are increased by concentration in the ashes obtained after incineration. On the other hand, anthropogenic radionuclides would be present due to the possible existence of sources (^137^Cs, ^60^Co, or ^241^Am) or the disposal of materials containing radionuclides from nuclear medicine (^131^I, ^99m^Tc, etc.). While some studies recommend radiation protection monitoring [5], others conclude that the presence of radionuclides in these wastes is at background levels [4]. Waste from residues with high organic content could concentrate 40K, as has been shown in biomass ashes in previous studies [6]. Additionally, studies on recycled metal waste from end-of-life vehicles (ELVM) showed the presence of natural and anthropogenic radionuclides (^137^Cs) at ambient levels [7]. However, the wastes that have been most extensively studied have been sewage treatment plant sludge (STPS) [8]. Sewage sludge can contain both naturally occurring radionuclides [9] and anthropogenic radionuclides such as ^137^Cs [10], although ^131^I and other radionuclides from hospitals (^99m^Tc, ^123^I, ^67^Ga, and ^201^Tl) are the more common radionuclides and most studies focus mainly on them [11,12].

These radionuclides used in nuclear medicine are characterized by a half-life in the order of days, which would reduce their danger due to decay. For this reason, sludges would be one of the fuels to be investigated to assess their potential hazard from a radiological protection perspective. It is also important to try to correlate the possible presence of radionuclides with the chemical composition of the waste used as fuel, using the same oxides as those used to determine the R-index. These correlations could provide valuable information for predicting which wastes would require monitoring from a radiological protection perspective. There are different databases on natural radionuclides in building materials and NORM waste [13,14]. Previous studies have established a correlation between these radionuclides and chemical composition by applying a principal component analysis [15,16,17]. However, no studies have yet correlated the radiological content of materials used in co-processing with their chemical composition.

In view of the above, the objective was to determine the radiological content of different wastes used for co-processing in the cement industry and its relationship with the chemical composition in the form of a percentage of metal oxides. To this end, the following partial objectives were proposed: (i) to classify the wastes according to their chemical composition using a principal component analysis and cluster analysis; (ii) to determine the activity concentrations of the raw materials by gamma spectrometry, estimating their presence in the final ashes; and (iii) to evaluate the annual effective doses of workers interacting with the ashes produced in the co-processing process.

## 2. Experimental Section

### 2.1. Sample Preparation and Chemical Characterization

**Figure 1:**
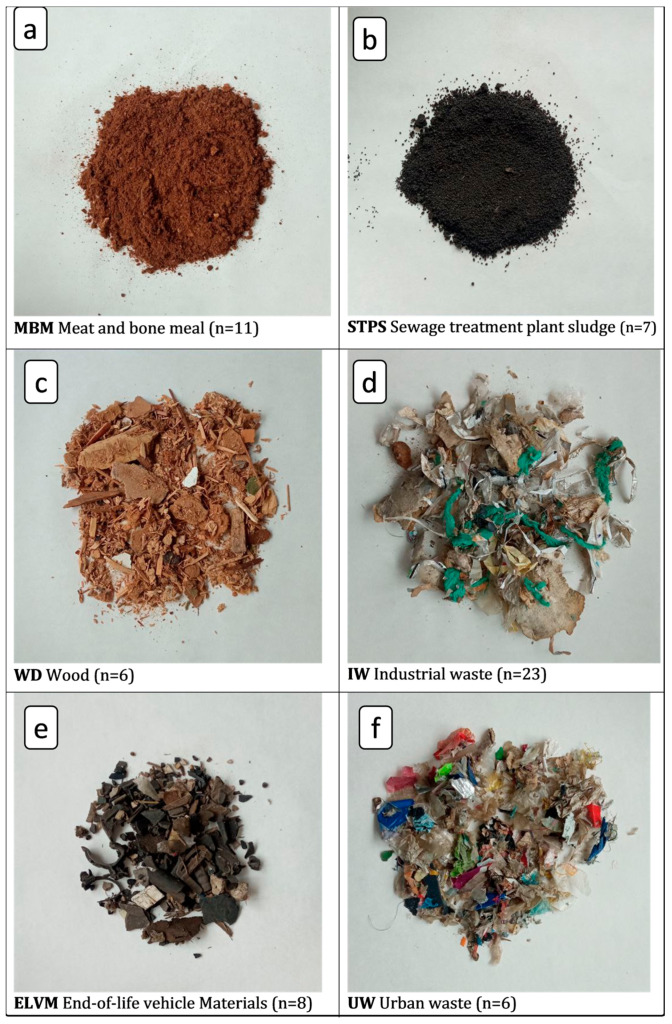
Types of waste used, together with its nomenclature and the number of samples of each type.

The number of samples studied was 63 from 6 different types of waste, chosen for their current and expected future consumption, as well as for their potential recycling rate [2]. They were supplied by LOEMCO. Figure 1 shows a picture of each waste type, indicating the nomenclature used in this study and the number of samples of each type.

Samples were labelled with the acronym of the residue and consecutive numbers up to the total number of samples, e.g., WD1, WD2, …, WD6.

A significant fraction of each sample was fractionated according to UNE-EN 15443:2011 [18]. The MBM, LD, and MD samples were quartered using groove splitters. CDRU, CDRI, and VFU samples were cut by cone stacking and subsequent quartering. The samples were subsequently weighed and oven-dried at 100 °C for 24 h to obtain 100–300 g aliquots. These samples were used to determine the activity concentration of the gamma emitters present (see Section 2.2). An equivalent amount was calcined in a muffle at 550 °C and the ashes were ground in a vibratory ring mill to a particle size of 100 μm. Finally, the samples were pelleted in a platinum crucible using a muffle (between 1200 and 1500 °C) with a flux (Li_2_B_4_O_7_) containing an oxidizing agent (LiNO_3_).

The chemical composition of the oxides in the samples was determined by X-ray fluorescence, following the recommendations of the UNE-EN 15410:2012 standard [19]. The equipment used was a Philips PW-1404 sequential spectrometer (LOEMCO, Madrid, Spain). The equipment had a rhodium anode X-ray tube, operated at a voltage of 30–50 kV and a current of 0.4–0.8 mA under vacuum conditions. Fluorescence spectra were acquired for 60–180 s per sample, and the equipment software allowed the quantification of the Al_2_O_3_, CaO, Fe_2_O_3_, K_2_O, MgO, Na_2_O, SO_3_, SiO_2_, and TiO_2_ contents present in the different ash samples of the recovered solid fuels investigated. Samples were measured only once, as the laboratory’s quality controls (based on ISO/IEC 17025:2017 [20]) ensure that a single measurement guarantees the reproducibility of the results.

The relationships between the oxides obtained and the types of samples were studied using the software RStudio version 2023.12.1, build 402. The principal component analysis (PCA) was performed using the libraries ‘FactoMineR’ and ‘Factoextra’. The criteria used to perform the PCA were the same as in previous works by the same authors [15,16]. The vectors of the variables selected were those with Kaiser–Meyer–Olkin (KMO) indices greater than 0.6, ensuring sufficient correlation between these variables [21]. The ‘Psych’ library was used to determine these parameters. The results obtained were represented in an HJ-Biplot graph [16], which allows the simultaneous plotting of the vectors determined from the variables studied (percentages of oxides), defining the components, with the scores obtained for the samples according to these vectors. Finally, the samples were grouped by the cluster analysis using the PCA results.

### 2.2. Measurement by Gamma Spectrometry

Among the 63 samples used, 13 representative samples of each residue type were selected and measured by gamma spectrometry, following the procedure described in Section 2.1; they were measured on four coaxial detectors: two extended range (XtRa, with an operating potential of up to +4000 V), one broad energy (BEGE with an operating potential of up to −4500), and one reverse electrode (REGe with an operating potential of up to +5000). All three detectors were characterized by Canberra, which allowed the calculation of counting efficiencies as a function of energy using the mathematical code LabSOCS [22]. The detectors were connected to a compact Mirion-Canberra DSA-LX electronics chain that contains the integrated high-voltage source, amplifier, and analog-to-digital converter, and allows connection to a computer (Figure 1) [23]. The spectra were acquired and analyzed using Genie 2000 software [24]. The samples were measured for a minimum of 80,000 s, and the backgrounds for 600,000 s. The spectra were analyzed taking into account the following parameters: (i) the peaks were located with a significance threshold of 3, (ii) the energy tolerance was ±2 keV for both samples and the background, (iii) the baseline was set as a step function to establish the regions of interest, and (iv) the fit of the photopeaks was Gaussian. The lower limit of detection was determined using the Currie criterion [25].

The laboratory where the measurements were performed is accredited based on the UNE-EN ISO/IEC 17025:2017 standard [20], which establishes periodic quality controls, as well as participation in intercomparative exercises. The above laboratory criteria ensured the reproducibility of the results from a single measurement.

**Figure 2:**
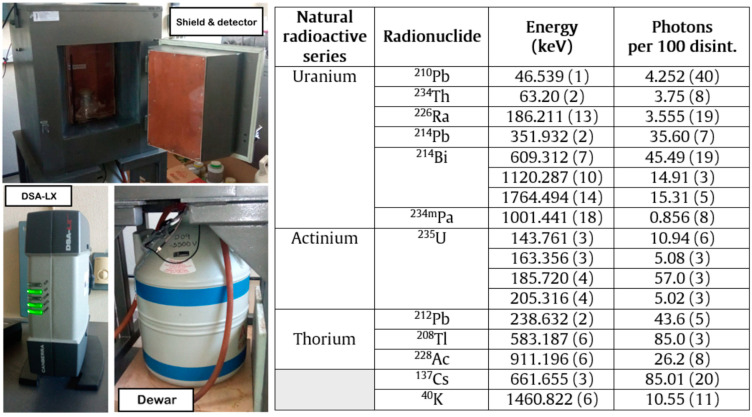
The schematic of the measurement system (DSA-LX, HPGe gamma detector, dewar vessel filled with liquid nitrogen, lead shielding, Tien and Cooper shielding for Pb X-ray removal) and photopic of the gamma emitters of the three natural radioactive series, and ^40^K and ^137^Cs from the fallout. The values of energy and photons per 100 disintegrations were taken from the tables of the Laboratorie National Henri Becquerel [26].

Figure 2 shows the photopeaks used for the determination of the activity concentration of the gamma-emitting radionuclides of the uranium, actinium, and thorium radioactive series, together with that of ^40^K of a natural origin and ^137^Cs of an anthropogenic origin [26]. The activity concentration of ^226^Ra was determined by removing the ^235^U interference from the 186 keV photopeak. Also, the interference produced by ^228^Ac in the 1460 keV photopeak was removed using the algorithm described in [27]. The true coincidence summing effect was corrected by the Peak-to-Total method using the total efficiency curve of the characterized detectors [28].

### 2.3. Calculation of the Natural Radionuclide Enrichment Factor of the Ashes Obtained from the Various Wastes after Use as Fuel

The activity concentrations of the different wastes studied were determined as de-scribed in Section 2.2. However, to assess the true radiological hazard and determine the effective doses, which will be described in Section 2.4, it is necessary to replicate the final state of the waste after combustion. Therefore, the final activity concentration was calculated considering the percentages of ash generated during the combustion process (CA). The enrichment factor in natural radionuclides (*F_e_*) with respect to the activity concentration of the uncalcined residues was determined by the following expression:

(2) Fe=An·1AC100

where $A_{n}$ is the activity concentration of radionuclide *n* (Bq kg^−1^), and *AC* is the ash content (%).

### 2.4. Assessment of the Effective Doses of the Residues Used for Workers

The doses from the final waste ash were assessed following the criteria set out in Radiation Protection 122 [29]. The doses were determined for the ‘small heap’ scenario for workers, where the total annual dose rate ($E_{A}$, μSv y^−1^) is given by expression (3) [30].

(3) $$E_{A}=E_{ext}+E_{inh}+E_{ing}$$

where $E_{ext}$, $E_{inh}$, and $E_{ing}$ are the annual effective dose rates from external gamma radiation, inhalation, and ingestion, respectively. These effective doses are determined for each of the radionuclides contained in the sample by the following expressions:

(4) $$E_{ext}=D_{ext}\cdot T_{e}\cdot A_{r}$$

(5) $$E_{inh}=D_{inh}\cdot T_{e}\cdot B_{r}\cdot C_{d}\cdot A_{r}$$

(6) $$E_{ing}=D_{ing}\cdot T_{e}\cdot R_{i}\cdot A_{r}$$

where $D_{ext}$, $D_{inh}$, and $D_{ing}$ are the dose conversion factors for external radiation, inhalation, and ingestion for each radionuclide; $T_{e}$ is the annual exposure time (1800 h y^−1^); $B_{r}$ is the respiratory rate (1.2 m^3^ h^−1^); $C_{d}$ is the dust concentration (2.0 × 10^−5^ kg m^−1^); $R_{i}$ is the ingestion rate (1.0 × 10^−5^ kg h^−1^); and $A_{r}$ is the activity concentrations of the radionuclide for which the effective dose is to be estimated [31].

## 3. Results

### 3.1. Relationship between Chemical Composition and Type of Waste

**Figure 3:**
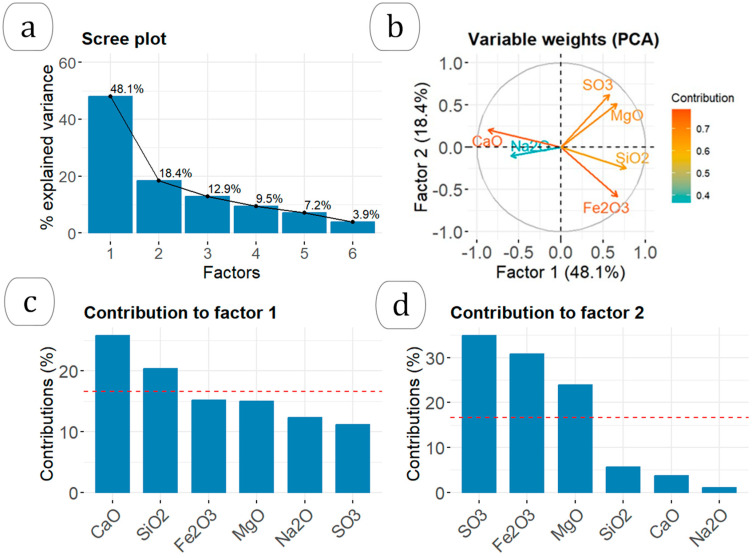
Results of the principal component analysis (PCA) of the residues according to the chemical composition obtained by XRF: (**a**) the scree plot with the % of variance explained according to the factors obtained, (**b**) correlations obtained between the different variables according to the 2 most important factors, (**c**) percentage contribution of the different variables to factor 1, and (**d**) percentage contribution of the different variables to factor 2.

Table 1 shows the percentage ranges of the oxides in each type of residue used in this study, along with the median values. Figure 3 shows the results from the principal component analysis (PCA) of the chemical composition of the residues in oxide form, as determined by XRF. Figure 3a presents the scree plot indicating that factors 1 and 2 account for 66.5% of the total variance. The PCA variable weight plot (Figure 3b) illustrates the correlations between the oxides of the various residues as obtained by XRF. The variables that met the criterion of having a Kaiser–Meyer–Olkin (KMO) value greater than 0.6 were CaO, Na_2_O, SO_3_, MgO, SiO_2_, and Fe_2_O_3_. However, the variables K_2_O, MgO, and TiO_2_ did not meet the criterion and were thus excluded from the PCA. The variables that showed correlation with one another were the CaO and Na_2_O oxides, MgO and SO_3_, and SiO_2_ and Fe_2_O_3_. Conversely, it was noted that the CaO and Na_2_O oxides were inversely proportional to the SiO_2_ and Fe_2_O_3_ oxides, forming a 180° angle between them. Additionally, these oxides were unrelated to the presence of SO_3_ and MgO, as evidenced by the 90° angle they formed (with a cosine of 0). The percentage contributions of the variables to factors 1 and 2 are depicted in Figure 3c,d. CaO and SiO_2_ had statistically significant contributions to factor 1, while SO_3_, Fe_2_O_3_, and MgO contributed to factor 2.

**Figure 4:**
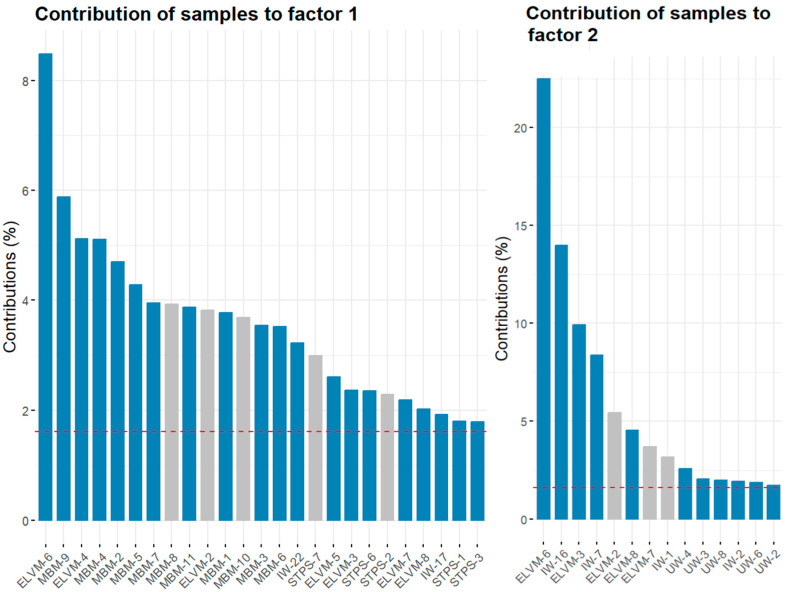
The contribution of the different samples to the factors with the highest GWP impact (48.1% and 18.4%, respectively). The dotted red line represents the expected average contribution (cutoff), with the columns shown for the two factors being those that exceeded this value.

Figure 4 shows the contributions of the individual samples to GWP factors 1 and 2. The grey bars represent the samples analyzed by gamma spectrometry. Factor 1 accounted for 75% of the ELVM residues, 100% of the MBM, 9% of the IW, and 71% of the STPS. Factor 2 accounted for 71% of the ELVM, 50% of the IW, 62% of the UW, and 14% of the STPS.

**Figure 5:**
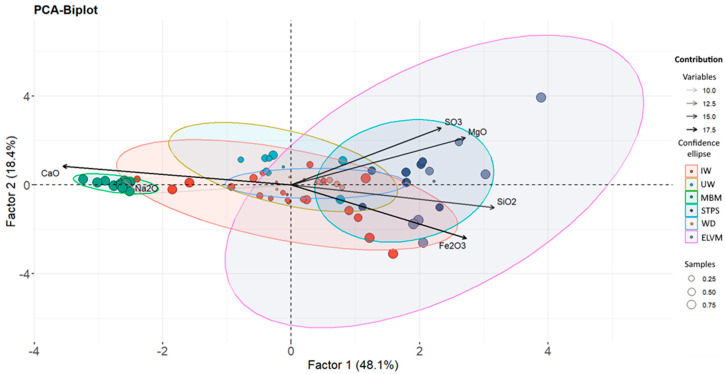
The HJ-Biplot graph presents the results of the principal component analysis along with the confidence ellipses for the different groups of residuals analyzed. The scores obtained for the various samples are shown in circular shapes whose sizes represent their contributions to the different components.

Figure 5 shows the HJ-Biplot graph representing the results obtained from the PCA. The confidence ellipses for the different groups of residuals are also presented, as well as the scores of the different samples for the two factors represented. The results indicate two statistically significant groupings: meat and bone meal (MBM) waste represented in green and sewage sludge (STPS) waste represented in blue. The remaining wastes are centered on the graph, indicating a lack of substantial correlation with the two factors obtained. On the other hand, the ELVM wastes, grouped in the fuchsia sector, although not centered, exhibited a pronounced dispersion.

**Figure 6:**
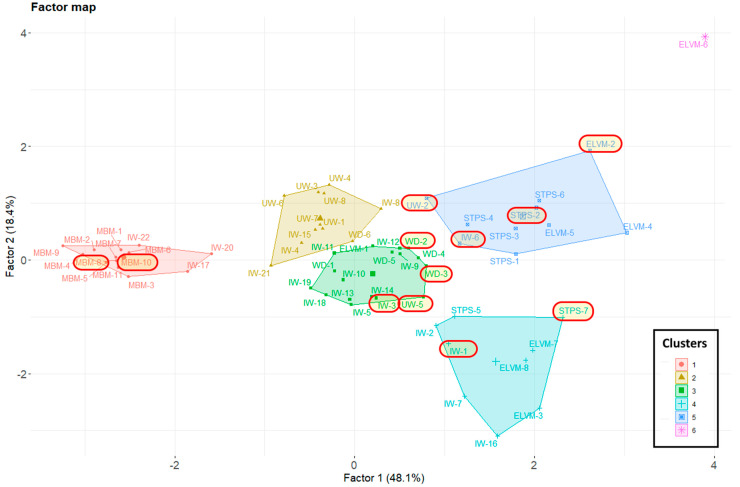
The cluster map created from the results of the principal component analysis. The total number of clusters resulting from the analysis was six.

The confidence ellipses in Figure 5 were generated based on residue type and not on the PCA results. Therefore, Figure 6 displays the groupings derived from the PCA results through the cluster analysis. Residues analyzed by gamma spectrometry are highlighted with a red box. The six groups obtained demonstrate a clear clustering of the meat meals (MBM), showing their high dependence on factor 1 (Na_2_O and CaO). The other residues exhibited greater heterogeneity and a lower correlation with the two factors identified, as they are centered in the graph. The wastes from sewage sludge (STP), although more dispersed than that from meat meal (MBM), are grouped together and show a relationship with both factors.

### 3.2. Activity Concentration of Natural Radionuclides in Different Wastes

Table 2 shows the activity concentrations of the natural radioactive series of uranium and thorium, ^40^K, and ^137^Cs in the 13 wastes selected and analyzed by gamma spectrometry. They were considered as reference levels for Portland cement as there are no world average levels for this type of waste. The world average level for radionuclides of the naturally occurring radioactive uranium and thorium series is 50 Bq kg^−1^, while the world average level for ^40^K is 500 Bq kg^−1^ [14,32]. Also, the value adopted for ^137^Cs was the fallout value in soils, which is 4 Bq kg^−1^ [33]. The activity concentrations of the ELVM, MBM, IW, UW, and WD wastes were on the order of or lower than the selected reference value. STPS sewage sludge was the only waste with values above 50 Bq kg^−1^ for ^234^Th and ^210^Pb. Also, sample IW-6 had an activity concentration for ^210^Pb also above 50 Bq kg^−1^. The activity concentrations of the radionuclides of the thorium series were all below 50 Bq kg^−1^. On the other hand, the activity concentrations of ^40^K were all below 500 Bq kg^−1^. Finally, the ^137^Cs activity concentrations were all below the limit of detection (LOD) determined from the Currie criterion [25].

### 3.3. Enrichment in Natural Radionuclides of the Ashes Obtained from the Different Wastes after Use as Fuels

**Figure 7:**
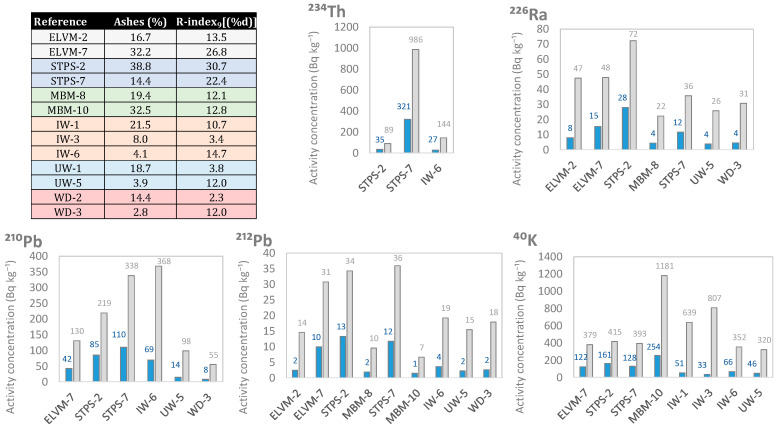
Increases in the activity concentration of radionuclides from the uranium (^234^Th, ^226^Ra, and ^210^Pb) and thorium (^212^Pb) series, along with ^40^K.

Figure 7 shows the increase in the activity concentration of the natural radionuclides from the uranium series (^234^Th, ^226^Ra, ^210^Pb), the thorium series (^212^Pb), and ^40^K when transforming the samples into ashes. The factors used to obtain the final activity concentration were calculated using expression 2, utilizing the ash percentages determined experimentally according to the procedure explained in Section 2.1. It can be seen that the ash percentages obtained were similar to the R-index_9_[(%d)] determined. The activity concentrations of ^226^Ra were estimated with ^226^Ra (determined from the 186 keV peak) and by ^214^Pb in cases where the ^226^Ra activity was lower than the limit of detection (LOD). The sewage sludge (STPS-2 and STPS-6) showed the highest increase in the activity concentration of ^234^Th, ^226^Ra, and ^210^Pb. IW-6 had the highest ^210^Pb activity concentration in the ash (368 Bq kg^−1^). On the other hand, MBM-10 obtained the highest concentration of ^40^K activity in the ashes (1181 Bq kg^−1^).

### 3.4. Effective Doses Received by Workers

Table 3 presents the effective doses obtained with expressions 3, 4, 5, and 6 using the activity concentrations estimated for the different radionuclides in the ashes of each waste type. The maximum value of the total annual effective dose rate was for the sludge wastes (STPS-2 and STPS-7). The wastes IW-1 and IW-3 only contributed external dose rates from gamma radiation, as shown in Table 2, since only ^40^K was detected. The annual effective dose rates for samples UW-1 and WD-2 could not be calculated because all activity concentrations of the studied radionuclides were below the limit of detection (L_D_).

## 4. Discussion

The results obtained confirmed the initial objective, since the two sludges analyzed by gamma spectrometry were those with the highest activity concentrations and therefore the highest total annual effective dose rate calculated from the estimation of the radiological content of the ashes.

The GWP showed two statistically significant groupings that were strongly influenced by chemical composition, meat meals and sewage sludge (Figure 3 and Figure 4). Meat meals were a priori together with woods the ones that could have the highest amount of ^40^K as supported by previous studies [34,35]. However, only sample MBM-10 obtained a ^40^K activity concentration of 254 ± 26 Bq kg^−1^. This result is consistent with the observed relationship between these materials and the amount of Na_2_O. Although K_2_O and Na_2_O performed equally well in the PCA, K_2_O had to be removed as it did not meet the criterion of a KMO > 0.6. Therefore, the higher concentration of ^40^K activity would be related to the presence of Na_2_O and K_2_O. The level of ^40^K, together with the high concentration produced in its transformation into ash (Figure 5), would result in annual effective dose rates between 0.0081 and 0.024 mSv y^−1^.

Sewage sludge had the highest activity concentrations of all the wastes analyzed. The activity concentrations were _238_U (^234^Th) > ^210^Pb > ^226^Ra for the uranium series radionuclides. Additionally, the ratio between ^226^Ra and ^232^Th was 1. These results align with those of other studies [12]. The HJ-Biplot graph demonstrated that the sludges had a higher concentration of Fe_2_O_3_ (Figure 3 and Figure 4). This suggests that the increased presence of uranium series radionuclides may be due to an association with iron compounds, as observed in other matrix types [15,16,30]. The ^40^K activity concentrations were also consistent with other studies, ranging from 136 Bq kg^−1^ to 497 Bq kg^−1^ [4,8]. The present results did not indicate the presence of ^137^Cs or ^131^I, isotopes typically found in such residues [10,12]. The transformation into ash likely results in the loss of these isotopes due to their boiling points, 671 °C for ^137^Cs and 184 °C for ^131^I. Consequently, authors like Carvallo [5] advise controlling the calcination process of these residues to prevent high inhalation doses. The present results, obtained without calcination, suggest no hazard from ^137^Cs. ^131^I would not pose a radiological problem after 56 days of storage, given its half-life of 8.0233 (18) days. The estimated annual effective dose rates for the ashes, calculated using Radiation Protection 122 (RP 122) [29], were in line with other studies, with values between 0.02 mSv and 1 [9]. Similarly, the annual effective dose rates were consistent with those from probabilistic models like RESRAD, which estimated them to be 0.03 mSv y^−1^ [36]. These doses are significantly lower than the natural annual radioactive background of 2.4 mSv y^−1^ [37], indicating no radiological risk under the present study conditions.

Finally, the ^210^Pb activity concentration results were 42 ± 12 Bq kg^−1^ and 69 ± 20 Bq kg^−1^ for ELVM-7 and IW-6, respectively. These activity concentrations were of the order of 50 Bq kg^−1^, which was the average value for building materials adopted as a comparative criterion. These wastes are also characterized by a higher Fe_2_O_3_ content, suggesting a possible relationship between chemical composition and ^210^Pb content, as observed for sewage sludge. However, the significant dispersion among the chemical compositions observed in the HJ-Biplot diagram necessitates further study of this type of waste. The ashes of these two materials would cause effective doses of 0.040 mSv y^−1^ and 0.016 mSv y^−1^, respectively. These effective dose rates are consistent with those found by other researchers, who reported 0.06 mSv y^−1^ for metal smelting facilities from vehicles [7].

## 5. Conclusions

The use of waste materials for the co-processing process in cement production does not pose risks from a radiation protection perspective. The annual dose rates estimated in this study for workers ranged from 0.0033 mSv to 0.092 mSv. The highest activity concentrations were observed in sewage ash, with ^238^U (^234^Th) at 321 ± 38 Bq kg^−1^ and ^210^Pb at 110 ± 14 Bq kg^−1^. ^210^Pb was quantified in samples from disused vehicle recycling and industrial waste. These radionuclides demonstrated a correlation with Fe_2_O_3_, which has also been observed in previous studies. The highest ^40^K activity concentrations were found in wastes with a higher organic matter content, such as meat and bone meal. The radioactive content of this type of waste was of a natural origin, as anthropogenic radionuclides like ^137^Cs were not detected in the wastes analyzed in this study. Future research could focus on the study of radionuclide leaching in ashes and also on those wastes with higher Fe_2_O_3_ content.

## Figures and Tables

**Table 1:** Percentage range of oxides of each of the residues used in this study, along with the mean obtained for each residue and oxide.

Oxide (%)	ELVM			STPS			MBM			UW			IW			WD		
	Min	Max	Med	Min	Max	Med	Min	Max	Med	Min	Max	Med	Min	Max	Med	Min	Max	Med
Al_2_O_3_	1.8	10.6	5.9	9.8	13.9	11.5	0.04	1.1	0.7	9.2	37.1	10.4	5.7	43.8	13.0	1.0	6.5	5.3
CaO	14.2	23.1	17.7	12.0	23.3	22.1	40.1	49.6	41.1	21.1	36.0	30.3	13.5	36.7	25.1	8.3	33.0	23.0
Fe_2_O_3_	1.7	19.4	7.8	9.6	18.9	11.0	0.2	3.8	0.8	1.9	7.6	2.5	1.0	20.1	3.7	0.7	6.2	3.8
K_2_O	0.2	1.4	0.4	1.5	2.7	1.9	1.6	6.3	4.4	0.6	2.7	2.2	0.8	9.3	1.3	1.7	15.0	3.5
MgO	2.3	11.6	3.2	2.6	5.0	3.8	1.1	1.7	1.4	2.7	4.6	3.1	1.6	4.1	2.1	2.3	4.0	3.6
Na_2_O	0.3	3.5	0.8	0.6	1.8	1.1	2.8	5.0	4.3	1.4	7.4	3.4	1.7	10.0	3.0	0.7	4.7	3.4
SO_3_	1.0	5.5	2.2	1.1	5.4	3.6	0.4	1.2	0.7	2.0	4.3	3.3	0.8	4.1	1.8	1.0	2.8	2.4
SiO_2_	11.6	39.3	32.4	15.1	25.0	21.8	1.0	7.3	5.7	15.4	31.6	18.4	7.3	40.1	23.9	6.2	38.9	27.2
TiO_2_	0.9	2.6	1.3	0.7	1.0	1.0	0.01	0.4	0.06	1.9	16.9	6.1	0.7	22.9	3.4	0.07	8.5	5.5

**Table 2:** Activity concentrations of the natural radioactive series of uranium and thorium, ^40^K, and ^137^Cs for the municipal waste analyzed by gamma spectrometry.

Reference	Uranium Series					Thorium Series			^40^K	^137^Cs
	^234^Th	^226^Ra	^214^Pb	^214^Bi	^210^Pb	^228^Ac	^212^Pb	^208^Tl		
ELVM-2	<7.1	7.9 ± 5.9	<1.9	<2.4	<8.9	<3.3	2.43 ± 0.41	0.91 ± 0.31	<11.4	<0.8
ELVM-7	<12.8	<24.3	15.4 ± 4.6	16.8 ± 2.0	42 ± 12	11.0 ± 5.4	9.9 ± 2.5	3.8 ± 1.6	122 ± 24	<1.5
MBM-6	<5.6	<7.6	4.33 ± 0.72	4.26 ± 0.86	<6.5	<2.5	1.85 ± 0.29	0.73 ± 0.22	<17.5	<0.6
MBM-10	<11.2	<21.6	<3.0	<2.7	<11.3	<5.0	1.42 ± 0.54	<1.1	254 ± 26	<1.3
STPS-6	321 ± 38	<16.0	11.6 ± 2.1	10.5 ± 1.1	110 ± 14	16.9 ± 1.9	11.7 ± 1.7	4.2 ± 1.2	128 ± 18	<0.8
STPS-2	34.6 ± 9.2	28 ± 13	14.7 ± 2.1	14.2 ± 1.9	85 ± 10	17.4 ± 2.3	13.3 ± 1.4	5.07 ± 0.82	161 ± 19	<1.2
IW-1	<19.7	<33.9	<7.5	<6.7	<18.0	<10.7	<3.7	<3.0	51 ± 13	<2.7
IW-3	<17.9	<27.7	<4.8	<4.8	<14.7	<6.3	<1.7	<2.2	33 ± 23	<1.9
IW-6	27 ± 14	<39.0	<5.2	<5.8	69 ± 20	<8.7	3.6 ± 1.1	<2.5	66 ± 18	<2.5
UW-1	<20.5	<36.6	<6.3	<7.7	<20.2	<11.9	<2.2	<3.2	<39.0	<2.9
UW-5	<7.3	<16.4	3.7 ± 1.7	6.0 ± 1.2	14.1 ± 8.8	<2.4	2.22 ± 0.54	<1.0	46 ± 16	<0.7
WD-2	<14.7	<33.5	<4.9	<5.1	<14.3	<7.0	<2.3	<2.2	<26.7	<1.3
WD-3	<5.5	<8.7	4.43 ± 0.89	4.5 ± 1.1	7.9 ± 3.5	<2.9	2.58 ± 0.36	1.12 ± 0.27	<12.4	<0.7

The uncertainties are quoted for a coverage factor of k = 2.

**Table 3:** Annual effective doses (mSv y^−1^) obtained from the estimated activity concentrations for the different radionuclides in the studied wastes. E_A_ is the total annual effective dose, E_ext_ is the external dose from gamma radiation, E_inh_ is the annual effective dose from inhalation, and E_ing_ is the annual effective dose from ingestion.

Sample	E_ext_ (mSv y^−1^)	E_inh_ (mSv y^−1^)	E_ing_ (mSv y^−1^)	E_A_ (mSv y^−1^)
ELVM-2	0.028 ± 0.012	0.0035 ± 0.0015	0.00052 ± 0.00022	0.032 ± 0.013
ELVM-7	0.032 ± 0.013	0.0071 ± 0.0029	0.00142 ± 0.00058	0.040 ± 0.016
MBM-6	0.046 ± 0.015	0.0131 ± 0.0042	0.00208 ± 0.00066	0.061 ± 0.020
MBM-10	0.0134 ± 0.0029	0.00220 ± 0.00047	0.000294 ± 0.000063	0.0159 ± 0.0034
STPS-6	0.0260 ± 0.0062	0.063 ± 0.015	0.00309 ± 0.00073	0.092 ± 0.022
STPS-2	0.0066 ± 0.0013	0.00137 ± 0.00027	0.000126 ± 0.000025	0.0081 ± 0.0016
IW-1	0.00329 ± 0.00042	-	-	0.00329 ± 0.00042
IW-3	0.0042 ± 0.0014	-	-	0.0042 ± 0.0014
IW-6	0.0036 ± 0.0013	0.0120 ± 0.0043	0.00063 ± 0.00023	0.0162 ± 0.0058
UW-1	-		-	-
UW-5	0.0175 ± 0.0077	0.0036 ± 0.0016	0.00074 ± 0.00033	0.0219 ± 0.0097
WD-2	-	-	-	-
WD-3	0.0189 ± 0.0058	0.0042 ± 0.0013	0.00088 ± 0.00027	0.0240 ± 0.0073

The uncertainties are quoted for a coverage factor of k = 1.

## Data Availability

Data are contained within the article.
